# Breathomics for diagnosing tuberculosis in diabetes mellitus patients

**DOI:** 10.3389/fmolb.2024.1436135

**Published:** 2024-08-13

**Authors:** Rong Xu, Ying Zhang, Zhaodong Li, Mingjie He, Hailin Lu, Guizhen Liu, Min Yang, Liang Fu, Xinchun Chen, Guofang Deng, Wenfei Wang

**Affiliations:** ^1^ Endocrinology Department, The First Affiliated Hospital of Gannan Medical University, Ganzhou, China; ^2^ Department of Endocrinology, The Third People’s Hospital of Shenzhen, National Clinical Research Center for Infectious Disease, Southern University of Science and Technology, Shenzhen, China; ^3^ Guangdong Provincial Key Laboratory of Regional Immunity and Diseases, Department of Pathogen Biology, Shenzhen University Medical School, Shenzhen, China; ^4^ Key Laboratory of Prevention and Treatment of Cardiovascular and Cerebrovascular Diseases, Ministry of Education, Gannan Medical University, Ganzhou, China; ^5^ Division Two of Pulmonary Diseases Department, The Third People’s Hospital of Shenzhen, National Clinical Research Center for Infectious Disease, Southern University of Science and Technology, Shenzhen, China; ^6^ National Clinical Research Center for Infectious Disease, The Third People’s Hospital of Shenzhen, Southern University of Science and Technology, Shenzhen, China

**Keywords:** breathomics, tuberculosis, diabetes mellitus, volatile organic compounds, XGBoost model

## Abstract

**Introduction:**

Individuals with diabetes mellitus (DM) are at an increased risk of *Mycobacterium tuberculosis* (Mtb) infection and progressing from latent tuberculosis (TB) infection to active tuberculosis disease. TB in the DM population is more likely to go undiagnosed due to smear-negative results.

**Methods:**

Exhaled breath samples were collected and analyzed using high-pressure photon ionization time-of-flight mass spectrometry. An eXtreme Gradient Boosting (XGBoost) model was utilized for breathomics analysis and TB detection.

**Results:**

XGBoost model achieved a sensitivity of 88.5%, specificity of 100%, accuracy of 90.2%, and an area under the curve (AUC) of 98.8%. The most significant feature across the entire set was m106, which demonstrated a sensitivity of 93%, specificity of 100%, and an AUC of 99.7%.

**Discussion:**

The breathomics-based TB detection method utilizing m106 exhibited high sensitivity and specificity potentially beneficial for clinical TB screening and diagnosis in individuals with diabetes.

## Introduction

Diabetes mellitus (DM), a rapidly increasing chronic illness on a global scale, comes with a multitude of complications and high mortality rates (Cole and Florez, 2020). Tuberculosis (TB) is the world’s second leading cause of death from a single infectious agent, after coronavirus disease (COVID-19) (WHO, 2022). Notably, 8 out of the top 10 countries burdened with high diabetes prevalence are also high-burden countries for tuberculosis. The prevalence of tuberculosis among diabetes patients can range from 0.38% to 14% (Al-Rifai et al., 2017). Poor control of blood sugar and diabetes significantly heightens the risk of contracting tuberculosis, with individuals having diabetes being up to four times more likely to develop active tuberculosis, negatively impacting tuberculosis treatment outcomes (Cáceres et al., 2022; Vaishya et al., 2024). The increasing prevalence of diabetes is anticipated to escalate the incidence of tuberculosis, posing a substantial threat to global public health when the two conditions coexist (Rehman et al., 2023; Zhao et al., 2023).

Presently, clinical diagnosis methods for TB revolve around sputum samples, including sputum smear microscopy, sputum culture, and GeneXpert testing, alongside non-sputum samples like bronchoalveolar lavage fluid examination and percutaneous tissue biopsy (Kontsevaya et al., 2023; Sodsri et al., 2023). Sputum smear microscopy testing often encounters a high rate of false negatives, while sputum culture, the primary method for diagnosing pulmonary tuberculosis, is hindered by low sensitivity and lengthy incubation periods (Broger et al., 2023). GeneXpert MTB/RIF (Xpert) achieved good performances in TB detection and drug resistance testing in the clinic and has been recommended by the WHO. However, it still requires good infrastructure and sputum samples (Chen et al., 2023). Moreover, bronchoalveolar lavage fluid examination and percutaneous tissue biopsy, as non-sputum sample detection methods, enhance the diagnostic accuracy but are intricate and invasive (Luo et al., 2023). Diabetes combined with tuberculosis (DM-TB) patients are more likely to experience delayed sputum culture conversion, treatment failure, and higher rates of relapse, which present a greater challenge to accurate diagnosis. TB diagnosis in individuals with diabetes is more prone to failure due to smear-negative results. Given the existing constraints in diagnosing tuberculosis among diabetes patients, there is a pressing need for innovative diagnostic technology to bolster current diagnostic capacities (Xiao et al., 2021).

Breath analysis, reflecting the body metabolism through volatile organic compounds (VOCs), presents over 3,500 known compounds, including acetone, isoprene, ethanol, ethane, and formaldehyde, closely tied to various diseases (Badola et al., 2023; Moura et al., 2023; Sun et al., 2023). VOCs in exhaled breath serve as disease biomarkers, and the detection process of VOCs is non-invasive and rapid, among other features. This method holds promise for early disease detection, treatment monitoring, and metabolic status evaluation, supported by numerous studies showcasing its potential. For example, the concentration of acetone in the breath of diabetes patients escalates with disease severity, while isoprene levels exhibit variations in diabetes, end-stage renal disease, lung cancer, and advanced liver fibrosis patients. Researchers have successfully identified VOC characteristics of lung cancer, colorectal cancer, breast cancer, and other diseases via exhaled breath analysis. Exhaled breath analysis has shown encouraging outcomes in diagnosing tuberculosis, respiratory inflammation, and COVID-19 (Fu et al., 2023; Paez et al., 2023; Savito et al., 2023; Hussain et al., 2024).

In this research, we utilized high-pressure photon ionization time-of-flight mass spectrometry (HPPI-TOFMS) to analyze the breath VOCs of diabetes and diabetes with tuberculosis patients to establish a diagnostic model based on breathomics for diabetes combined with tuberculosis, subsequently validating it on clinical datasets.

## Materials and methods

### Study design and participants

**FIGURE 1:**
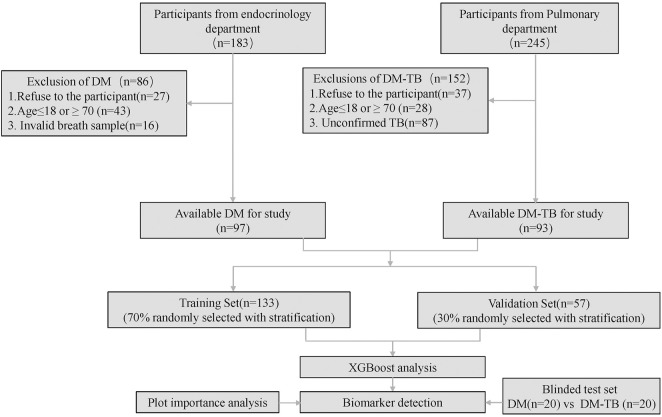
Flowchart of participant recruitment.

This cross-sectional study was conducted at the Third People’s Hospital of Shenzhen from August 2022 to September 2023. The study was approved by the Ethics Committee of the Third People’s Hospital of Shenzhen (number: 2022-012). By the end of the study period, we recruited a total of 190 participants, i.e., 93 diabetes patients and 97 diabetes combined with tuberculosis patients. The participant enrollment flow is illustrated in Figure 1. All participants provided informed consent.

Inclusion criteria for DM were as follows: participants aged 18–70 years; following the WHO 1999 diagnostic criteria for diabetes and the 2011 WHO recommendation to use HbA1c for diabetes diagnosis in countries and regions with the necessary conditions, with a diagnostic threshold of HbA1c ≥ 6.5%.

Inclusion criteria for patients with DM-TB: patients with diabetes aged 18–70 years; clinical and radiological evidence of pulmonary tuberculosis, with positive microbiological evidence (positive sputum culture for *Mycobacterium tuberculosis* or positive GeneXpert result); and anti-TB treatment not initiated or started less than 2 weeks.

The clinical diagnosis and collection of breath samples were overseen by physicians, ensuring expertise in both areas. Separate researchers, who were blinded to clinical data and other test outcomes, handled the detection of VOCs and machine learning modeling. Furthermore, the physicians were intentionally kept unaware of the breath test results to maintain objectivity. Demographic and clinical information for all participants was meticulously collected and organized, with a comprehensive summary provided in Table 1.

**TABLE 1:** Demographic characteristics of participants.

	Training set			Test set		
	DM + TB (N = 65)	DM (N = 68)	*p*-value	DM + TB (N = 28)	DM (N = 29)	*p*-value
Age						
Median	54 (27–70)	48 (23–70)	< 0.001	51 (25–70)	46 (21–70)	< 0.001
<35 (%)	2 (3.1)	13 (19.1)	0.68	1 (3.6)	6 (20.7)	0.47
≥35 (%)	63 (96.9)	58 (80.9)	0.019	27 (96.4)	23 (79.3)	0.023
Sex						
Male (%)	54 (83.1)	43 (63.2)	0.012	23 (82.2)	18 (62.0)	0.141
Female (%)	11 (16.9)	25 (36.8)	-	5 (17.8)	11 (38.0)	-

### Exhaled breath collection

All breath samples were collected following a predefined protocol and analyzed within a 24-h timeframe. The sampling setup consisted of a single-use gas nozzle and a sampling bag crafted from polyether–ether–ketone (PEEK) material. To ensure standardized procedures and reduce the impact of daily dietary variations, strict sampling requirements and protocols were implemented in this study. Initially, for inpatient participants, sampling was conducted during a subsequent visit, with individuals advised to prepare beforehand by refraining from smoking, alcohol consumption, or eating within an hour leading up to the sampling session. Subsequently, the participants were instructed to rinse their mouths with purified water immediately before sampling to mitigate the influence of dietary factors and smoking. Lastly, all samples were collected in a consistent environment to minimize the impact of external variables. The participants were instructed to take a deep nasal breath and fully exhale into the sampling bag, which had a volume exceeding 1.2 L.

### Breath sample detection

The HPPI-TOFMS system utilized in this study comprises a vacuum ultraviolet (VUV) lamp-based HPPI ion source and an orthogonal acceleration time-of-flight (TOF) mass analyzer for the detection and analysis of breath samples. VOCs with an ionization potential lower than 10.6 eV were directly ionized in the ionization region. The gas-phase breath samples were directly introduced into the ionization region through a 250-μm inner diameter, 0.60-m-long capillary from the sampling bag. The TOF ion spectra were recorded using a time-to-digital converter, with mass spectra accumulated over 60 s. Mass spectrum peaks with m/z < 350 were detected using the HPPI-TOFMS system for each exhaled breath sample.

### XGBoost model construction

We selected the eXtreme Gradient Boosting (XGBoost) classification model with a gbtree kernel to distinguish DM and DM-TB. XGBoost is a machine learning algorithm that uses decision tree ensembles to build predictive models. It calculates the importance of features to eliminate unnecessary features and improve model performance and interpretability. All participants were randomly divided into two groups: 70% for constructing the model and the remaining 30% for blinded model testing. Hence, 93 DM patients and 97 DM-TB patients were randomly chosen as the discovery dataset. Through 100 iterations of 7:3 randomization, the discovery dataset was split into a training subset and an internal validation subset. Sensitivity, specificity, accuracy, positive predictive value, and negative predictive value were calculated. Receiver operating characteristic curves were generated, and the area under the curve (AUC) was calculated to evaluate the diagnostic model.

### Performance evaluation and statistical analysis

Orthogonal partial least-squares (OPLS)-DA analysis was conducted to assess the overall differences between the two groups. Then, differential VOCs were selected based on the variable importance in projection (VIP) values in OPLS-DA analysis, fold change, and *p*-value in univariate analysis. A volcano plot was generated to visualize the differential VOCs. For parametric data, Student’s *t*-test was used for pairwise comparisons between the two groups. Analyses of data were conducted using GraphPad Prism 9 software (San Diego, CA). A *p*-value ≤ 0.05 was considered statistically significant.

## Result

### HPPI-TOF-MS detection

**FIGURE 2:**
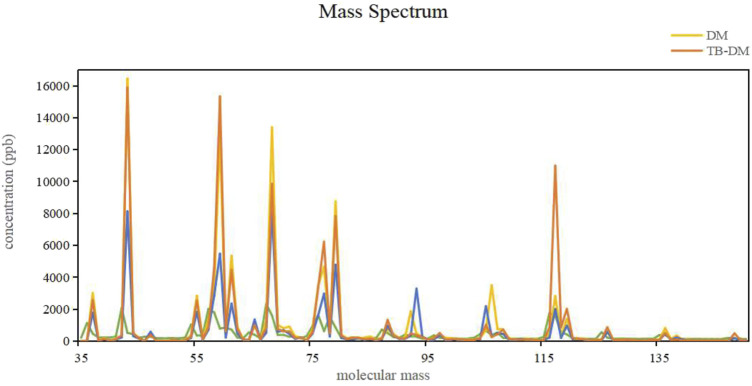
VOC detection spectrum of the participants.

For each participant, the detection range is from a molecular weight of 0–345, avoiding the molecular weight of the ion source, including the molecular weights where 95% of the concentration values can be detected. Eventually, the molecular weight range included is from 39 to 150, with a molecular weight interval of 1. To reduce model complexity, the number of input features was decreased while retaining more feature information; all concentration values detected within the molecular weight interval of 1 were stacked to maximize the preservation of the original data features and the number of features. A total of 111 features were obtained, and the calculated stable wave form average value was calculated and considered the concentration of the VOCs. The spectrum of detection is shown in Figure 2. Endogenous VOCs that had stable and low-noise waveforms were used for the model construction.

### Evaluation of the XGBoost model and feature importance

**FIGURE 3:**
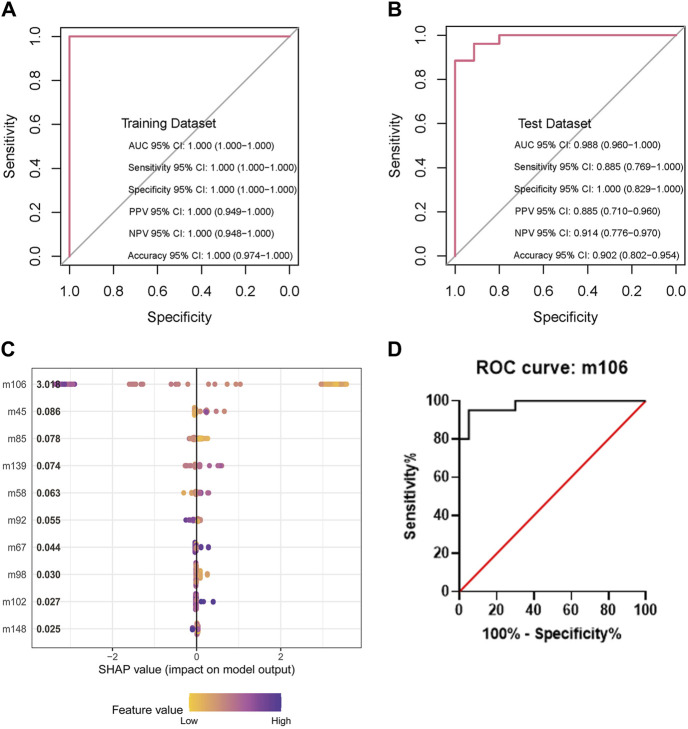
Evaluation of the XGBoost models and feature importance. **(A,B)** Sensitivity, specificity, and accuracy of the model in the training and test datasets. **(C)** Feature importance of the model in the dataset by SHAP. **(D)** Receiver operating characteristic curve analysis based on VOC m106; m106 represents m/z = 106.0.

The XGBoost model attained a sensitivity of 100%, a specificity of 100%, an accuracy of 1, and an AUC of 1.0 (95% confidence interval [CI]: 1.0 to 1.0) in the training dataset. The test dataset achieved a sensitivity of 88.5%, a specificity of 100%, an accuracy of 0.902, and an AUC of 98.8% (95% CI: 0.960 to 1.0). These indicators demonstrated the performance of the model. The AUC reflected the robustness of the models. The evaluation of the models is shown in Figures 3A, B. Various popular machine learning (ML) models such as random forest (RF), support vector machine (SVM), logistic regression (LR), XGB, and decision tree (DT) were utilized as classifiers to differentiate between DM-TB and DM (Table 2).

**TABLE 2:** Performance of different ML models for TB detection in the test dataset.

Dataset	Model	Sensitivity	Specificity	PPV	NPV	Accuracy	AUC
Test (n = 57)	MARS	0.885	0.967	0.958	0.906	0.929	0.981
	RF	1.000	1.000	0.966	0.970	0.968	1.000
	SVM	0.717	0.783	0.800	0.793	0.796	0.795
	KNN	0.962	0.964	0.962	0.964	0.963	0.963

Feature importance refers to the contribution of each VOC and radiology report to the prediction score. The SHapley Additive exPlanations (SHAP) method was used to evaluate the XGBoost model. It indicates the classification capability of each feature. The most important features for the whole set were m106 (represents m/z = 106.0), as shown in Figure 3C. The data achieved a sensitivity of 93%, a specificity of 100%, and an AUC of 99.7% (95% CI: 0.993 to 1.0) based on m106 only (Figure 3D).

### VOC m106 was a potential biomarker for detecting DM-TB

**FIGURE 4:**
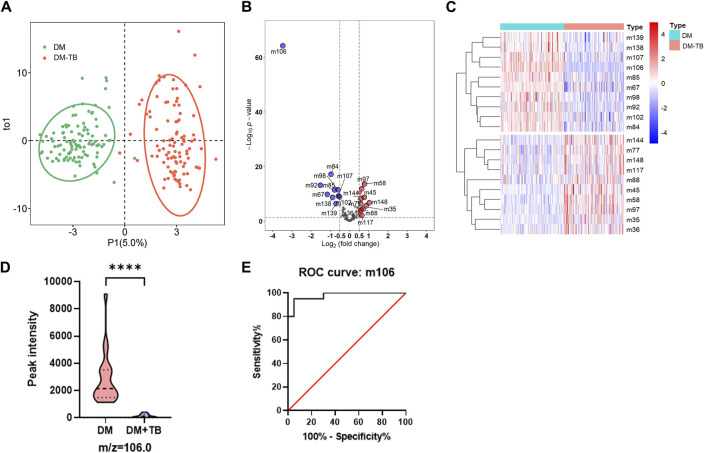
VOC m106 was a potential biomarker for detecting DM-TB. **(A)** OPLS-DA model for the DM and DM-TB groups. **(B)** Volcanic map of differential VOCs for the DM and DM-TB groups. **(C)** Clustering on differential VOCs for the DM and DM-TB groups. **(D)** VOC m106 concentration between the DM and DM-TB groups in the blinded test set. **(E)** Receiver operating characteristic curve analysis based on VOC m106 in the blinded test set. The differences among groups were compared using an unpaired *t*-test. ^****^ *p* ≤ 0.001.

To further validate the significant feature of VOC m106 to differentiate DM-TB patients from DM patients, an OPLS-DA model was used to avoid overfitting and evaluate the statistical significance of the model. The OPLS-DA score plot showed clear separation between the DM group and the DM-TB group based on the concentration of all VOCs (Figure 4A). According to the heatmap and the volcano plot, the content of m106 significantly decreases in the DM-TB group (Figures 4B, C). A blinded test set including DM (n = 20) and DM-TB patients (n = 20) was established to validate m106 concentration using HPPI-TOF-MS in these two groups. The data show that the content of m106 indeed significantly decreases in the DM-TB group and achieved distinguished efficacy (Figures 4D, E).

## Discussion

Screening for tuberculosis in diabetes patients can help identify tuberculosis symptoms early, leading to prompt diagnosis and treatment and reducing the transmission of tuberculosis in healthcare facilities and communities (Zhou et al., 2023). However, compared to isolated pulmonary TB, clinical symptoms of DM-TB are typically atypical. Patients may present with milder symptoms but more pronounced radiological findings. Currently, there are several limitations in sputum-based tuberculosis diagnostic technologies such as inaccuracy, high costs, and complexity of operation (Xu et al., 2023).

Research on breathomics mainly focused on respiratory system diseases. With the emergence of various detection technologies, the application scope has gradually expanded to include diabetes, cancer, kidney disease, liver disease, cognitive impairments, and other conditions (Ibrahim et al., 2021; Freddi and Sangaletti, 2022; Khoubnasabjafari et al., 2022). In our previous research, we explored the diagnostic significance of utilizing HPPI-TOF-MS for breathomics data analysis in the context of PTB within a sizable study group; exhaled breath samples were collected from 518 PTB patients and 887 controls and tested. Machine learning algorithms based on different VOCs were used for breathomics analysis and PTB detection. The differences between the two groups did not include the substance with m/z = 106. This may indicate its specificity in detecting TB-DM from another perspective (Fu et al., 2023). This method is non-invasive and does not require sputum samples. From breath sampling to obtaining test results, it takes only approximately 5 min, greatly improving the diagnostic efficiency (Meng et al., 2021). Our findings indicate that the breathomics approach we proposed shows promise as an effective diagnostic tool for screening TB for clinical applications.

Diabetes and tuberculosis are both common clinical diseases that can coexist; yet, there is currently no research on exhaled VOCs related to diabetes combined with tuberculosis. In this study, we explore the diagnostic value of breathomics data detection for DM-TB. The results demonstrated that the XGBoost model performs well in distinguishing DM-TB individuals and DM patients with high sensitivity and specificity of 88.5% and 100%. During the feature importance analysis, VOC m106 displayed the highest feature value with a score of 3.018. However, the complexity of breath gas composition and limitations to HPPI-TOFMS technology make it difficult to confirm the specific substance for m/z = 106. Furthermore, the substance with m/z = 106 may also have multiple forms, which makes it difficult to identify using standard reference materials. We can only use m/z = 106 as a biomarker to distinguish between DM and TB-DM. This is the current research challenge and limitation. Receiver operating characteristic curve analysis achieved a sensitivity of 93%, a specificity of 100%, and an AUC of 99.7% based on VOC m106. In the blinded test set, the data also show that the content of VOC m106 indeed significantly decreases in the DM-TB group and achieved well-distinguished efficacy. VOC m106 was a potential biomarker for detecting DM-TB.

However, there are still some limitations to this study. HPPI-TOF-MS primarily quantitatively analyzes VOCs, with m/z and peak intensity serving as surrogate indicators of concentration. Chemical composition analysis is still required for confirmation. Further studies are needed to confirm specific breath biomarkers for DM-TB and clarify their metabolic pathways.

## Data Availability

The raw data supporting the conclusion of this article will be made available by the authors, without undue reservation.
